# Using inhibition of the adipogenesis of adipose-derived stem cells *in vitro* for toxicity prediction

**DOI:** 10.1016/j.mex.2021.101515

**Published:** 2021-09-14

**Authors:** Ana Paula Ressetti Abud, Ariane Caroline Campos Paschoal, Crisciele Kuligovski, Rodrigo Rêgo Barros Caruso, Bruno Dallagiovanna, Alessandra Melo de Aguiar

**Affiliations:** a Rede de Plataformas Tecnológicas FIOCRUZ - Bioensaios com Métodos alternativos em Citotoxicidade, Rua Professor Algacyr Munhoz Mader, 3775, Instituto Carlos Chagas, FIOCRUZ Paraná, Curitiba, PR 81350-010, Brazil; b Laboratório de Biologia Básica de Células-Tronco, Rua Professor Algacyr Munhoz Mader, 3775, Instituto Carlos Chagas, FIOCRUZ Paraná, Curitiba, PR 81350-010, Brazil; c Grupo Boticário, Pesquisa and Desenvolvimento, Avenida Rui Barbosa, 4110, São José dos Pinhais, PR 83055-320, Brazil; d Laboratório de Ciências e Tecnologias Aplicadas à Saúde, Rua Professor Algacyr Munhoz Mader, 3775, Instituto Carlos Chagas, FIOCRUZ Paraná, Curitiba, PR 81350-010, Brazil; e Current Address: Laboratório de Biologia Molecular e Sistêmica de Tripanossomatídeos. Rua Professor Algacyr Munhoz Mader, 3775, Instituto Carlos Chagas, FIOCRUZ Paraná, Curitiba, PR 81350-010, Brazil

**Keywords:** Stem cells, Differentiation, Cytotoxicity

## Abstract

In vitro stem cell models are used as alternatives to animal models and are important tools for cytotoxicity studies. Researchers can determine the effects of test substances on human cells by evaluating cell viability and differentiation. Here, we describe an in vitro model to quantify adipogenesis based on the Nile red staining of specific lipid droplets and the emission of basic lipids from human adipose tissue-derived mesenchymal stromal cells (AD-MSCs) in the presence of test substances. This assay allows for the prediction of toxicity based on the inhibition of adipogenesis in vitro in a 96-well format. The differentiation of a progenitor cell into a specialized cell, the adipocyte, is easy to monitor and quantify, making this a simple assay. The fluorescence staining of nuclei and lipid droplets is measured after 14 days of cell differentiation to determine cell number and assess cell differentiation using high-content imaging analysis, thus allowing for the identification of chemicals that impact differentiation. We also describe a protocol to assess adipocyte differentiation by fluorescence intensity using a multiplate reader.

• Researchers can utilize the protocol described here for many purposes to evaluate in vitro adipogenesis.
• *With this method, it is possible to reduce the use of animals.*

## Graphical abstract

*AD-MSCs adipogenesis-inhibition assay in culture.* Schematic diagram showing the adipogenic inhibition assay. In this method, AD-MSCs are induced to differentiate and are maintained for 14 days in the presence of different concentrations of test chemicals. Therefore, it is possible to visualize the effects of the substances during the cell differentiation process. The results can be obtained by a high-content imaging system or microplate reader.

**Figure:**
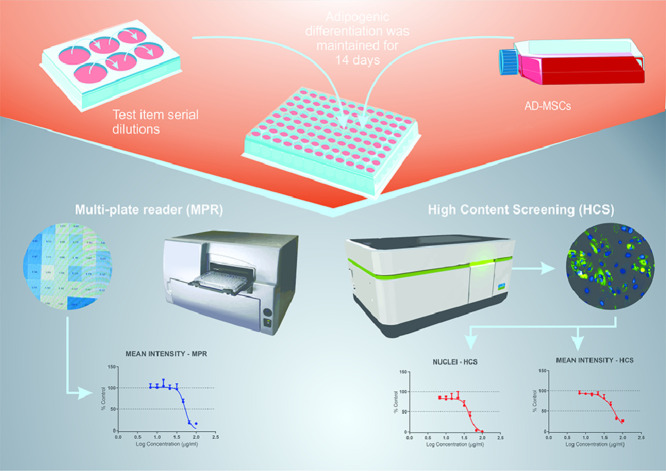

Specifications Table

**Table:** 

Subject Area:	Pharmacology, Toxicology and Pharmaceutical Science
More specific subject area:	*Alternative method to animal testing*
Method name:	*Adipogenesis Inhibition for Toxicity Prediction*
Name and reference of original method:	*N.A.*
Resource availability:	*N.A.*

## Method introduction

Currently, there are several different methodologies to assess the toxicological effects of test chemicals on human cells and animal tissues *in vitro* and *ex vivo*. Researchers use 2D cell culture in many assays that have already been validated; however, these methods are limited, as they use nonhuman lineage cells that may have chromosomal anomalies or primary cells that have limitations in the number of passages and the variability between batches [1]. In this context, human stem cells have been explored as a valuable *in vitro* system for toxicological studies [2] as an alternative to using animals or traditional *in vitro* methods. Due to their advantages of self-renewal capability and cell differentiation, stem cells can be very useful for the evaluation of toxicological mechanisms. These specific characteristics may provide additional strategies and applicable data for human toxicity predictions and to improve sensitivity [3,4]. In addition, it is possible to carry out tests on different biological samples to evaluate biological variability [4], [5], [6], [7]. Moreover, stem cells derived from tissue removed during surgery, specifically liposuction procedures [4,8], are of great interest because they represent disposable material, are easy to handle, and do not require a wide variety of supplements to culture [9].

Given the advantages of stem cells as alternatives to animal models and the need to reduce animal usage, we describe a system that can be used to quantify adipogenesis *in vitro* and apply an adipogenesis-inhibition protocol to estimate toxicity adequately; we also predict the best-fit starting dose for animal testing with the acute toxic class (ATC) method [10].

The protocol described here first involves the cultivation of human adipose tissue-derived mesenchymal stromal cells (AD-MSCs) [11]. Next, we describe the cell plating process, the induction of adipogenesis with dexamethasone, insulin, indomethacin and 3-isobutyl-1-methylxanthine (IBMX) and the treatment of the cells with the test substances. We performed Nile red staining to observe the specific lipid droplets and the emission of the basic lipids. In parallel, a test plate with sodium dodecyl sulfate (SDS) was prepared and used as a test-performance control. We describe the analysis using a high-content imaging system or hybrid plate reader to evaluate toxicity, and the graphical abstract shows the protocols. We aimed to determine the effects of test substances by generating a dose-response curve and calculated the concentration of a test compound that could decrease the endpoint by 50% (IC_50_ value) for the inhibition of adipogenesis. Then, we predicted the 50% lethal dose (LD_50_ value) to estimate the starting dose for the ATC method [10,12].

In summary, the protocol described here can be useful for the evaluation of cytotoxicity during cell differentiation processes *in vitro*.

## Method details

### Basic protocol: Adipogenic differentiation inhibition assay

AD-MSCs are plated and maintained under ideal culture conditions. Cell cultures then undergo adipogenic differentiation during exposure to the test substance. Users should have tested and prepared working solutions of the chemicals of interest (see also Support Protocol). All assays include a separate plate containing diverse SDS concentrations, which serve as the external positive control of adipogenesis inhibition. After 14 days of induction and treatment, users fix and stain the AD-MSCs with 4′,6-diamidino-2-phenylindole, dilactate (DAPI) and Nile red solution. Then, the users scan the plates via high-throughput microscopy (although this can also be performed using a plate reader). After data acquisition, we describe how to perform the necessary statistical analyses. The user will then be able to evaluate the toxicity of test chemicals and estimate the starting dose for an acute toxicity test (ATC).

### Protocol steps

#### Cell plating

1. Grow and maintain AD-MSCs in maintenance media for cell culture (see Reagents and Solutions).

*The cells should be cultured in an incubator maintained at 37°C ± 1°C, 90% ± 10% humidity and 5% ± 1% ambient CO_2_.*

2. AD-MSCs should be subcultured when they reach approximately 80–90% confluence.

• Remove the maintenance medium from the cell culture.
• Wash the cells with 10 ml of PBS for each 75-cm^2^ culture flask.
• Add 2 mL of the trypsin/EDTA solution and incubate for 3 min in an incubator at 37°C ± 1°C, 90% ± 10% humidity and 5% ± 1% ambient CO_2_.
• Observe the cells under an inverted phase contrast microscope to visualize the disruption of the monolayer.
• Add 4 ml of the maintenance medium for cell culture to the disrupted cells to inhibit trypsin enzymatic action.
• Take a sample of the cell suspension and perform the Trypan Blue Exclusion Test of Cell Viability as previously described (Strober, 2015), ensuring at least 80% viability. While counting the cells, keep the original cell suspension on ice to avoid cell clumping.

*Trypan blue solution permeates the membranes of dead cells, which incorporate blue staining, while live cells with an intact membrane remain bright and do not incorporate blue staining. This method ensures that the experiments are only carried out under conditions with cell viability greater than 80%.*

3. Make a cell solution with a concentration of 3.5 × 10^4^ cells/ml in maintenance medium.

4. Add 100 µl of the cell solution per well to 96-well plates (i.e., *3.5 × 10^3^ cells per well).*

*The user should use plates compatible with a high-content imaging system.*

*Each 96-well plate receives a total volume of 9.6 ml of cell suspension at 3.5 × 10^4^ cells/ml. To determine the total volume of cell suspension that will be required for the assay, it is necessary to multiply the total volume by the number of plates in the test. To ensure that a sufficient amount of cell suspension is prepared, the user should produce at least 3–5 ml of additional cell suspension.*

5. Maintain the cells at 37°C ± 1°C and 90% ± 10% humidity in a 5% ± 1% CO_2_ atmosphere for 24 h ± 2 h.

### Adipogenesis inhibition assay

6. Discard the medium by placing the plates upside down on sterile gauze to remove residual culture medium.

7. Add maintenance medium for cell culture (100 μl/well) to the wells in columns 1 and 12. These columns will represent the negative controls of the experiment (see Fig. 2).

8. Add adipogenic differentiation induction medium [2x] (50 μl/well) to the wells in columns 2 to 11 (Fig. 2).

9. Add 50 µl/well of the test item dilution medium to columns 2 and 11 to prepare the positive control (differentiated and untreated cells) (see Fig. 3).

10. Add serial dilutions of the test item to columns 3 through 10, from the highest to the lowest concentration, to create a gradient (see Fig. 3). Add 50 µl from well 1 of the 6-well plate to each well in column 3 of the 96-well plate (the contents of well 2 should be added to the wells in column 4, well 3 to the wells in column 5, well 4 to the wells in column 6, well 5 to the wells in column 7, well 6 to the wells in column 8, well 7 to the wells in column 9, and well 8 to the wells in column 10). Fig. 6 shows a schematic representation of the test dilution.

*If you start with the lowest concentration of the test chemical (i.e., column 10), you can reuse the tips.*

11. Place the plates in an incubator at 37°C ± 1°C, 90% ± 10% humidity, and 5% ± 1% CO_2_ for 14 days.

Every 3 or 4 days, the user should renew the culture medium and test items (i.e., on days 3, 7, and 10 of adipogenic induction).

*NOTE: All assays include a plate with the SDS external control. Support Protocol - Preparation of test items and testing of external positive control serial dilution describes the preparation of this item.*

*NOTE: Remove the plates from the incubator one at a time to avoid extended times outside of the incubator.*

*NOTE: For the adipogenesis inhibition assay, 4 channels of the 8-channel micropipette should be used, and the content of each well of the 6-well plate should be transported to the 96-well plate in a volume of 50 µl/well.*

### Cell fixation

After 14 days of induction and treatment, the AD-MSCs are fixed with a 4% paraformaldehyde solution, as described below.

12. Remove the medium of the plates by discarding the media, and dry the plates on sterile gauze.

13. Wash the plate twice with PBS [1x] prewarmed to 37°C (200 µl/well).

14. Add 4% paraformaldehyde solution (50 µl/well) and incubate the plates at room temperature for 10 min.

15. Wash the plates twice with PBS [1x] prewarmed to 37°C (200 µl/well).

*Here, you can either proceed to the staining protocol or stop the protocol at this time. If you decide to stop, keep the PBS [1x] in the plate and store it at 2–8°C until staining for a maximum period of 5 days.*

### Cell staining

16. Wash the plate twice with PBS [1x] prewarmed to 37°C (200 µl/well).

17. Stain the cells with Nile red working solution (50 µl/well) and incubate for 30 min, protected from direct light.

*The Nile red working solution is the result of a 1:1000 dilution of the stock solution of the dye (1 mg/ml) in PBS [1x].*

18. Wash the plate twice with PBS [1x] prewarmed to 37°C ± 1°C (200 µl/well).

19. Stain the cells with DAPI solution (50 µl/well) and incubate at room temperature for 10 min, protected from direct light.

20. Wash 3 times with PBS [1x] and store the plate with the buffer on the cells at 2–8°C until data acquisition.

*NOTE: The plates can be stored for up to 15 days before imaging.*

*If the user has a high-content imaging microscope and an HCI system, they should proceed to the next step.*

*If users do not have access to a high-content imaging system, they can use a plate reader (BioTek®, Winooski, VT, USA or similar) for data acquisition. The user should obtain the specific signal for basic lipid droplets stained with Nile red, a specific marker of the adipogenesis-specific positive signal at wavelengths of 450–500 nm excitation and higher than 528 nm emission* [13], [14], [15] *. After the user obtains the data, they should analyze the data relative to the mean intensity as described in the Statistical Analysis.*

### Data acquisition by high-content imaging microscopy

21. Turn on the equipment and establish the reading parameters as suggested below. The high-content imaging system setup will identify the cell number using the proper excitation and emission channels to detect nuclei stained with DAPI (e.g., excitation 355–285 nm, emission 430–500 nm or other suitable adjustments); to create a cytoplasm mask to identify the cell cytoplasm based on Nile red staining and the emission of the total cellular lipids in the red channel (e.g., excitation 530–560 nm, emission 570–650 nm; suitable for Alexa 546); and to observe the specific lipid droplets based on Nile red staining and the emission of the basic lipids (e.g., excitation 460–490 nm, emission 500–550 nm; suitable for Alexa 488), as well as a brightfield channel. Fig. 4 summarizes the required channels and suggested setup.

22. Perform an additional setup of the equipment following the manufacturer's instructions for general data acquisition. Adjust the focus or autofocus and define suitable acquisition parameters for each equipment model, including the intensity or power of the light source and time of exposition in milliseconds, among others.

23. Choose the 20 × magnification objective if possible; other magnifications may be evaluated if 20 × is not available.

*NOTE: If 20x is not available, the user should choose a magnification that can provide the correct identification of cell nuclei and the discrimination between the positive and negative controls on the test plate as explained below.*

24. Choose 13 alternate fields or 25 grouped fields for scanning for each well.

*NOTE: Thirteen to 25 fields per well in 8 wells for each treatment condition are normally adequate* (Fig. 5)*. This choice is critical because it is advisable to select the fewest number of fields to reduce not only the time required to read the plates but also the size of the files generated.*

*25.* Read the plate following the instructions of the equipment and save the data. Data can be stored, and the user can perform the image analysis later.

### Image analysis setup by high-content imaging microscopy

26. Identify the nuclei. Based on nuclear staining by DAPI, identify the nuclei using channel 1 and select nuclei that do not touch the borders to remove the border objects and avoid analyzing the same cell twice.

27. Identify the cytoplasm. Identify the cytoplasm based on channel 2, red staining for Nile red dye.

28. Calculate the cytoplasmic intensity properties for channel 3, including the specific lipid markers of adipogenesis and green staining for Nile red dye.

29. Select the adipose-positive cell (Adipo+) population. Based on the cytoplasmic intensity of the markers in channel 3, define cutoffs between the populations that are negative and positive for adipogenesis. Fig. 8 shows an example. In this example, the line that is shown in the dot plot graph represents the cutoff between the (-) and (+) cell populations. The x-axis shows the object number, and the intensity of the cytoplasmic staining is shown on the y-axis. The images shown in red are the negative population, and those shown in green are the positive population used for the readouts. Calculate the intensity and morphological properties of the selected Adipo+ cells.

30. Select the results for nuclei number, % Adipo+ cells, sum area of the Adipo+ cells, and sum intensity of the Adipo+ cells to construct the results table.

31. Save the image setup analysis.

32. Apply the image analysis setup to the plate.

33. Export the data to a format compatible with spreadsheet software (e.g., Microsoft Excel).

34. Analyze the data as recommended in the Statistical Analysis section (see Basic Protocol).

*NOTE: The user can save the image acquisition setup and the image analysis setup and apply them to other plates. The user should verify the focus distance at each acquisition. The user should verify the intensity cutoff between the negative and positive controls at each plate analysis.*

*NOTE: Depending on the equipment, the user can perform a default analysis to quantify the cell number and cytoplasm markers to evaluate adipogenesis. As an example, in* Fig. 8 *, we have shown an Operetta CLS Analysis workflow using Harmony 4.8. However, the user can apply and test other workflows for each laboratory and piece of equipment.*

### Statistical analysis

35. Transfer the readout from the high-content imaging system to spreadsheet software (e.g., Microsoft Excel).

36. Assess the assay quality by quantifying the difference between the values of the positive and negative control wells in each plate for each of the following analyzed readouts, including nuclei number, % Adipo+ cells, sum area of the Adipo+ cells, and sum intensity of the Adipo+ cells.

MAD = median (|Xi – median(X)|)

$$SSMD_{robust}=\frac{median\;C^{+}-\;median\;C^{-}}{\sqrt{MAD^{2}C^{+}-\;MAD^{2}C^{-}}}$$

where the median absolute deviation (MAD) is the median of the absolute differences between each individual value from the data and the median of the data, and C+ and C− are the positive and negative controls, respectively.

|SSMDrobust| ≥ 3 indicates a probability higher than 95% that the value from one control group is greater than a value from another control group.

If |SSMDrobust| ≥ 3, proceed as follows.

37. Exclude the minimum and maximum values from each of the 8 columns with the tested concentrations and from each of the 2 columns with a positive control.

38. Calculate the mean and standard deviation for each group.

39. Convert the values above into percentages of the mean of the adipogenesis-positive control.

40. With statistical software (e.g., GraphPad Prism®), perform nonlinear regression with the percentages above by applying the sigmoidal dose-response curve (variable slope) with four parameters (known as the Hill equation or four-parameter logistic curve) with a constrained bottom parameter (bottom = 0). This equation will fit the data to a sigmoidal curve.

41. Calculate the corrected IC_50_ values using the parameters of the nonlinear regression. Apply the following equations:

$$log\;IC_{50}\left(corrected\right)=log\;EC_{50}-\frac{log\left(\frac{top\;-bottom}{y-bottom}\;-1\right)}{Hill\;Coefficient}$$

$$IC_{50}\left(corrected\right)=10{\;}^{log\;IC50\;\left(corrected\right)}$$

where IC_50_ is the concentration that produces 50% toxicity; EC_50_ is the concentration that produces a response midway between the top and bottom responses; top is the maximum viability (%); bottom = 0; Y = 50 (i.e., 50% response), and Hill coefficient (or Hill slope) is a measure of the slope of the sigmoidal curve.

Another parameter provided by the nonlinear regression is R^2^, which is a measure of the fit of the data points to the sigmoidal curve.

**Fig. 1:**
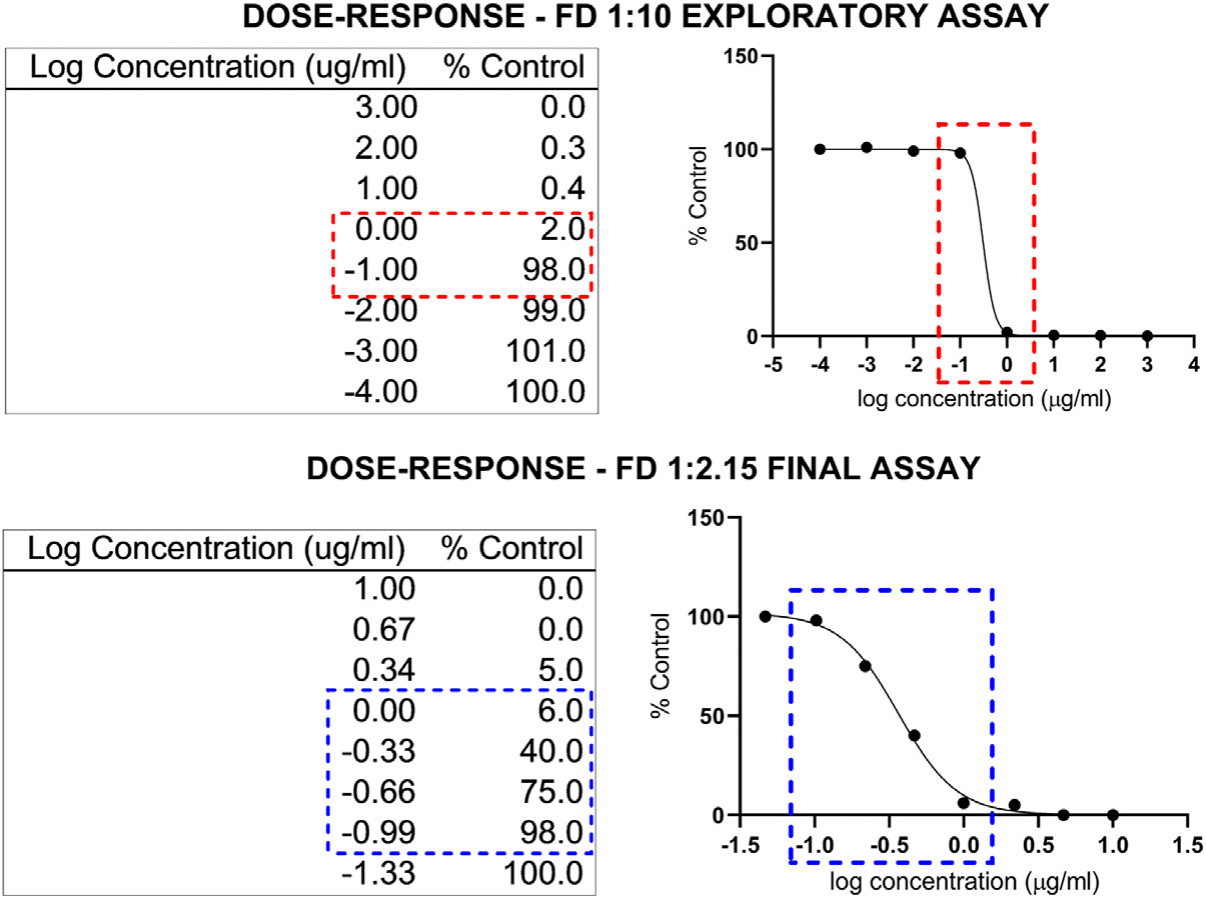
Exploratory and final assays. In this example, an exploratory test was conducted with a DF of 10. In the upper panel, the gap between the concentrations log -1 and log 0 (0.1–1 µg/ml) shows that the 0% and 100% effects are very close in the curve (we can see almost 100% and 0% of the control, respectively). To better resolve this dose-response curve, we performed a second assay with a serial dilution with a DF of 2.15, including 4 values between log -1 and log 0 and other values below and above 50%.

As an example, the Hill equation (nonlinear regression of data) from Fig. 1 (final assay) yields the following parameters:

Bottom = 0
Top = 101.8
IC_50_ = 0.3664
LogIC_50_ = -0.4360
Hill slope = -2.221
R^2^ = 0.9971
  $$\text{log}\;IC_{50}\left(\text{corrected}\right)=-0.4360-\frac{log\left(\frac{101.8}{50}\;-1\right)}{-2.221}=-0.436-\frac{0.01536}{-2.221}=-0.4291$$
  $$IC_{50}\left(\text{corrected}\right)=10{\;}^{-0.4291}=0.37\;\mu g/\text{ml}$$

**Fig. 2:**
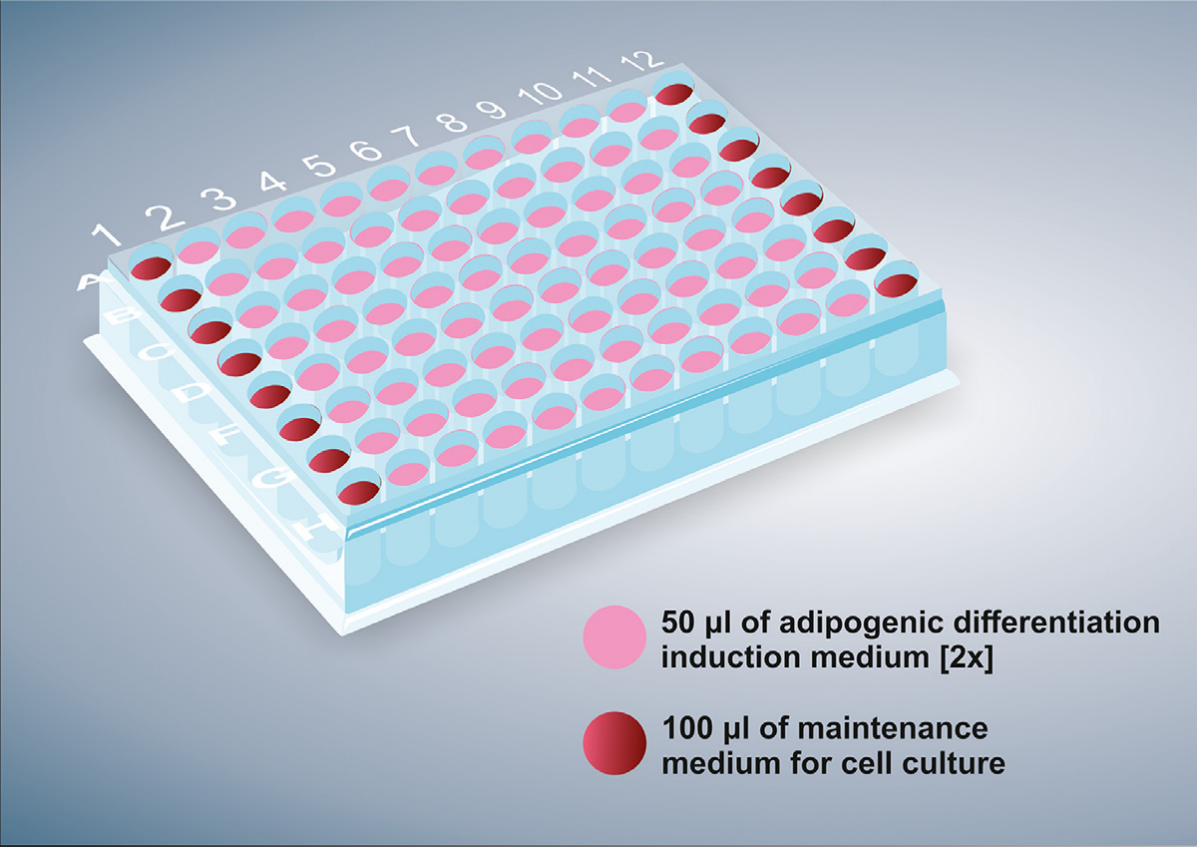
Schematic representation of the medium exchange. Moments before exposure of the test substance serial dilutions to the cells, 50 µl per well of adipogenic differentiation induction medium (2X) (columns 2–11) and 100 µl per well of maintenance medium for cell culture (columns 1 and 12) were added to the cells in 96-well plates. Columns 1 and 12 correspond to the negative control of the assay (only cells and maintenance medium).

**Fig. 3:**
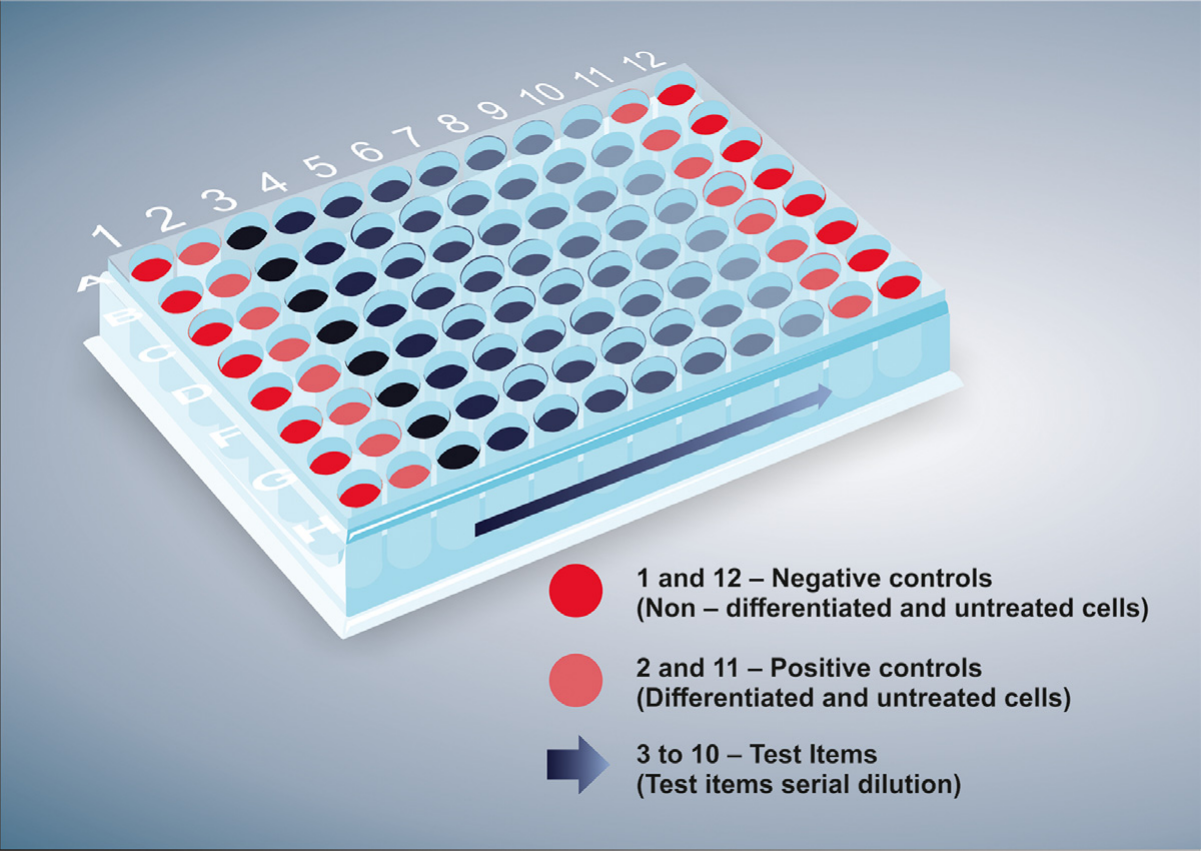
Schematic representation of the 96-well plate after distribution of all of the control and test solutions (the negative control, positive control, and eight test substance concentrations).

**Fig. 4:**
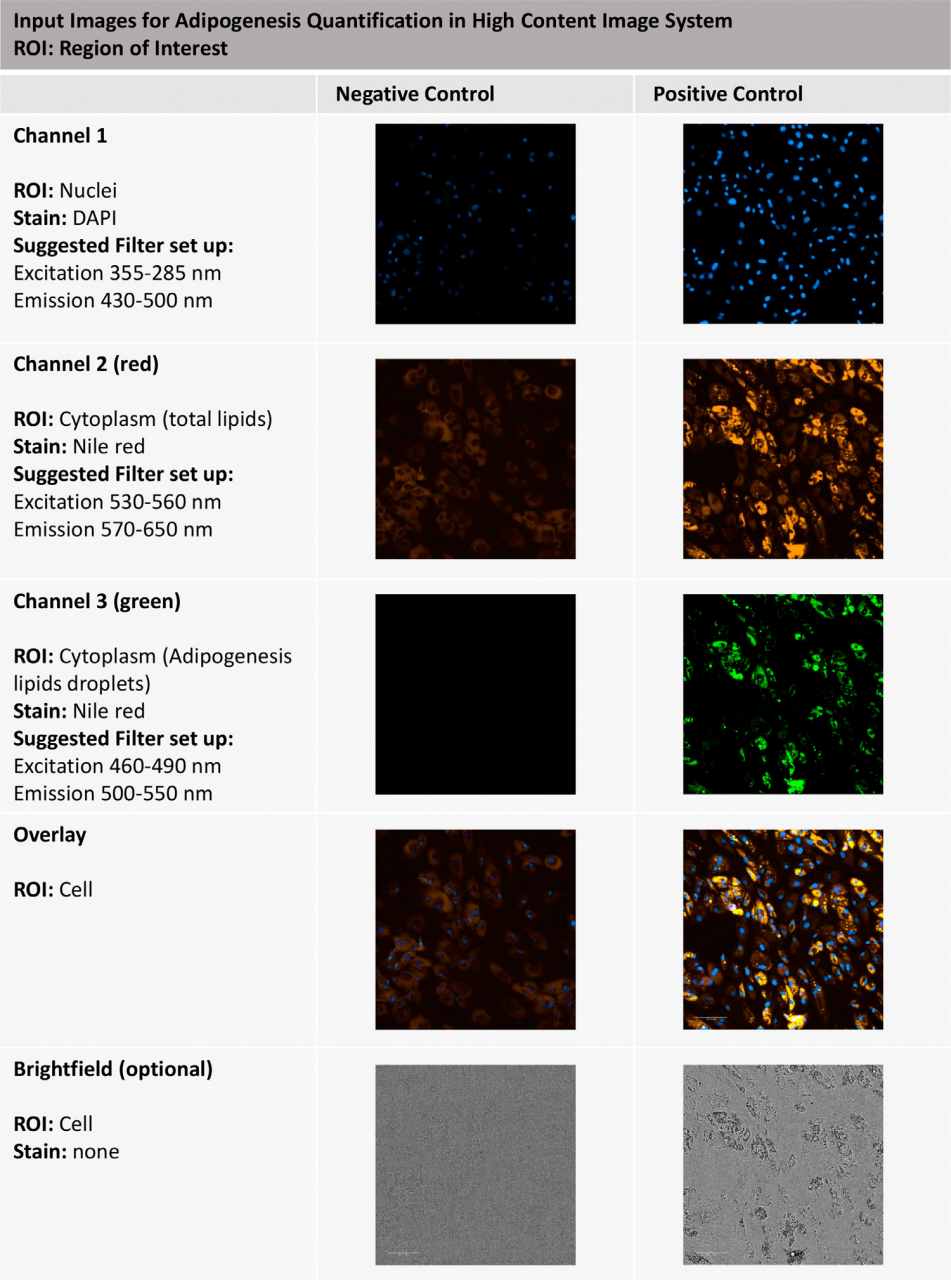
Input images for adipogenesis quantification with a high-content imaging system setup. Negative and positive controls are shown in the three fluorescence channels necessary for image analysis for adipogenesis quantification. The brightfield channel is shown as an optional channel to be included in the acquisition setup. ROI: region of interest.

**Fig. 5:**
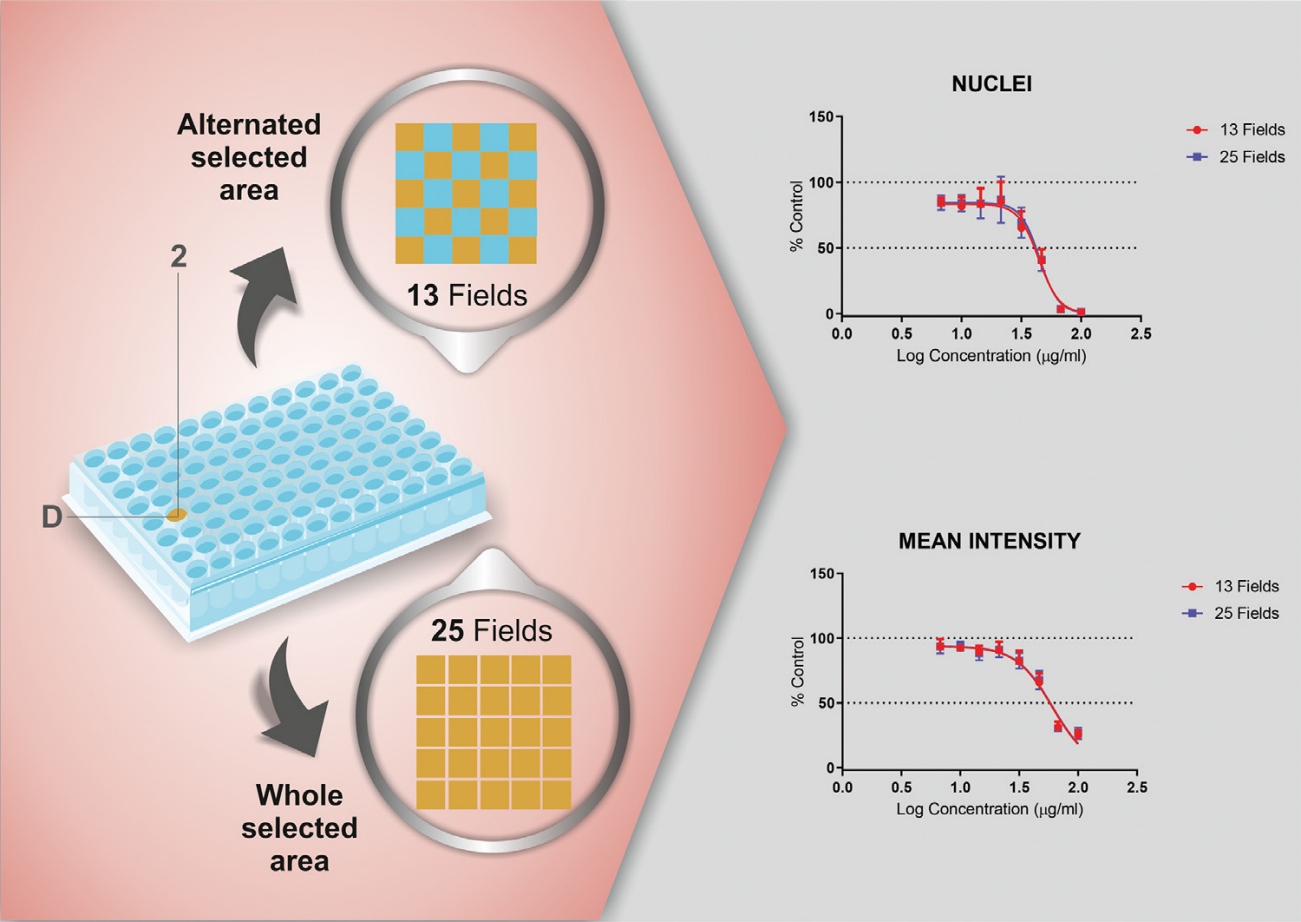
Plate reading strategy. Thirteen alternate fields or 25 grouped fields were scanned for each well. This step is critical to select the fewest number of fields to be scanned to reduce the reading time and generated file size.

42. Calculate the IC_50_ of the tested substance as the geometric mean of all assays.

$$\left(\text{geometric}\;\text{mean}\right)=n\sqrt{x1x2\ldots xn}$$

where x1, x2, … xn are the IC_50_ values calculated from individual assays and n is the number of considered assays.

### Estimating the starting dose for the acute toxicity test (ATC)

43. Use the IC_50_ values to predict the LD_50_ and GHS category of the tested substances. These predicted LD_50_ values are suitable for estimating the starting dose for the ATC (Table 2).

44. Apply the following formulas [16,17]:

Registry of Cytotoxicity (RC) rat-only weight regression: log LD_50_ (mg/kg) = 0.372 log IC_50_ (μg/ml) + 2.024

RC rat-only millimole regression: log LD_50_ (mmol/kg) = 0.439 log IC_50_ (mM) + 0.621

### Acceptance criteria of the test

45. Follow the previously established assay evaluation criteria (OECD, 2010; Abud et al., 2019) to accept or reject the assay based on the parameters % Adipo+ cells, sum area of the Adipo+ cells, and sum intensity of the Adipo+ cells:

- Sigmoidal curve R^2^ value ≥ 0.85.
- At least one calculated cytotoxicity value > 0% and ≤ 50% and at least one calculated cytotoxicity value > 50% and <100% present in the Hill equation model fit.
- SDS values that correctly predict the GHS category (toxicity category 4).
- |SSMDrobust| value ≥ 3.

*NOTE: Cell differentiation is the principal assay parameter. The user should not consider the nuclear parameter for assay exclusion.*

### Support protocol: Preparation of test items and testing of external positive control serial dilutions

Here, we describe how to prepare the dilutions of the tested substances in the adipogenesis inhibition assay described in Basic Protocol. Serial dilutions of the test substances should be prepared minutes before they contact the cells plated in the 96-well plates. Below, the serial dilution of hydrosoluble and nonhydrosoluble items are shown. An additional plate with an external control test substance, SDS, should be included in each assay to monitor test performance. We show the serial dilution of the SDS below.

### Protocol steps

#### Hydrosoluble test item

**Fig. 6:**
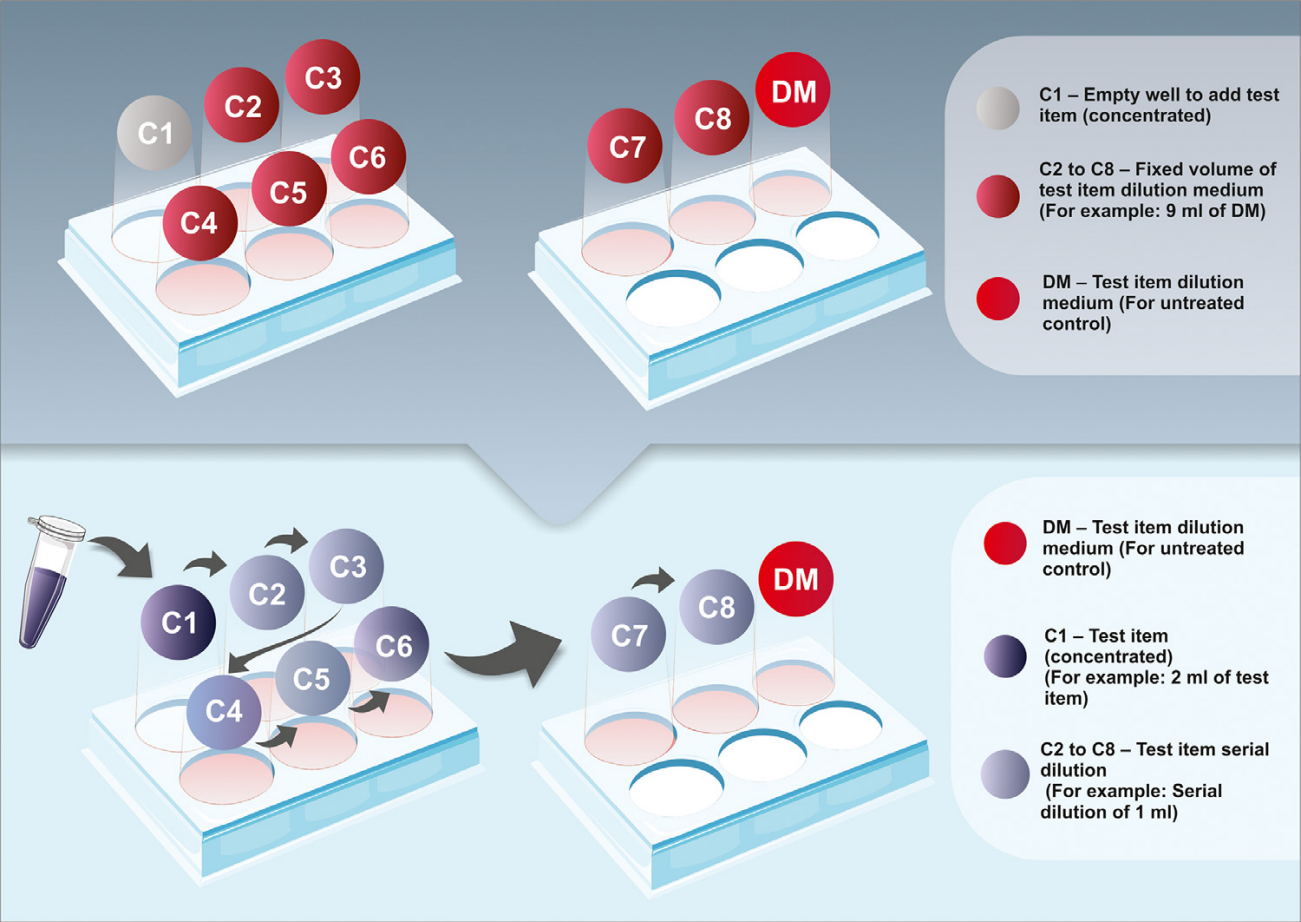
Schematic representation of the serial dilution of the test substance in the plates, including the 8 concentrations to be tested and DM used as the vehicle control. This sample representation of the serial dilution uses the 1:10 logarithmic factor, and there is always a fixed volume of 9 ml of the test item DM for every 1 ml of the test item solution. A serial dilution strategy is applied by transferring 1 ml of the more concentrated dilution, e.g., C1 to C2, mixing, and then continuing throughout the plate to C8.

46. A planning document must be generated, describing, in detail, the concentrations and preparation of the chemical substances to be tested. It is necessary to plan the evaluation of each test item, as shown in Annex A.

47. Before starting the dilutions, the user must weigh and/or dilute the chemical substances to obtain a working solution [2x] based on the highest concentration to be tested. This information must be present in the planning document (Annex A).

48. Vortex the solution for 1 to 2 min to ensure solubilization.

49. Use two sterile 6-well plates, where only 9 wells will be used. Number the wells from 1 to 8; the 9^th^ well will contain dilution medium (DM) without the test substance that should be used in the positive control columns.

50. To well 1, add the previously prepared working test solution [2x].

51. To wells 2 through 8, add a fixed volume of the test item DM, according to the predetermined logarithmic dilution factor indicated in the planning document (Annex A).

52. Initiate the serial dilution from well 1, which contains the working solution [2x]. Sequentially transfer a fixed volume of the test substance from wells 1 through 8 (Fig. 6 – Bottom part). Perform adequate homogenization in each well to properly mix the test substance with the previously deposited medium.

53. Once the serial dilution of the test substance is ready, return to step 10 from the basic protocol.

#### Nonhydrosoluble test item

**Fig. 7:**
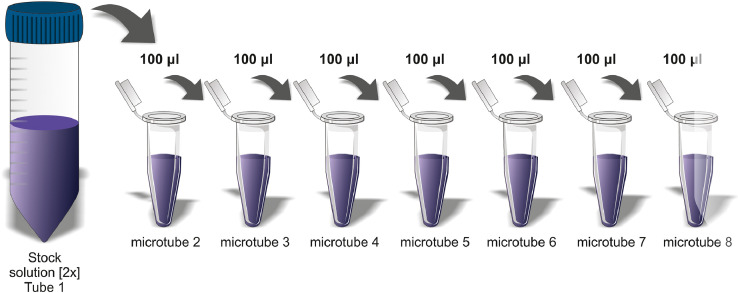
Schematic representation of a nonhydrosoluble test item serial dilution, including the 8 concentrations to be tested. The DF should be determined by exploratory tests. In this example, a DF of the logarithmic factor 1.78 was applied. All microtubes contained a fixed volume of 78 µl of solvent (in this case DMSO) for every 100 µl of serially diluted test substance.

54. A planning document must be generated, describing, in detail, the concentrations and preparation of the chemical substances to be tested. It is necessary to plan the evaluation of each test item, as shown in Annex B.

55. Before starting the dilutions, the user must weigh and/or dilute the chemical substances to obtain a working solution [2x] based on the highest concentration to be tested. This information must be present in the planning document (Annex B).

56. Vortex the solution for 1 to 2 min.

57. Prepare seven microtubes, and number them 2 to 8. Perform the serial dilution in these microtubes.

58. Add the required volume of dimethyl sulfoxide (DMSO) (the solvent used) to each microtube, according to Annex B. The example shows the volume associated with logarithmic factor 1.78, but other volumes can be added according to the logarithmic factor used.

59. Initiate the serial dilution from the stock solution [2x]. Sequentially transfer a fixed volume of the test substance until reaching microtube 8. Perform adequate homogenization in each microtube to properly mix the added test substance with the previously deposited solvent (DMSO). Fig. 7 shows a schematic of this process.

**Fig. 8:**
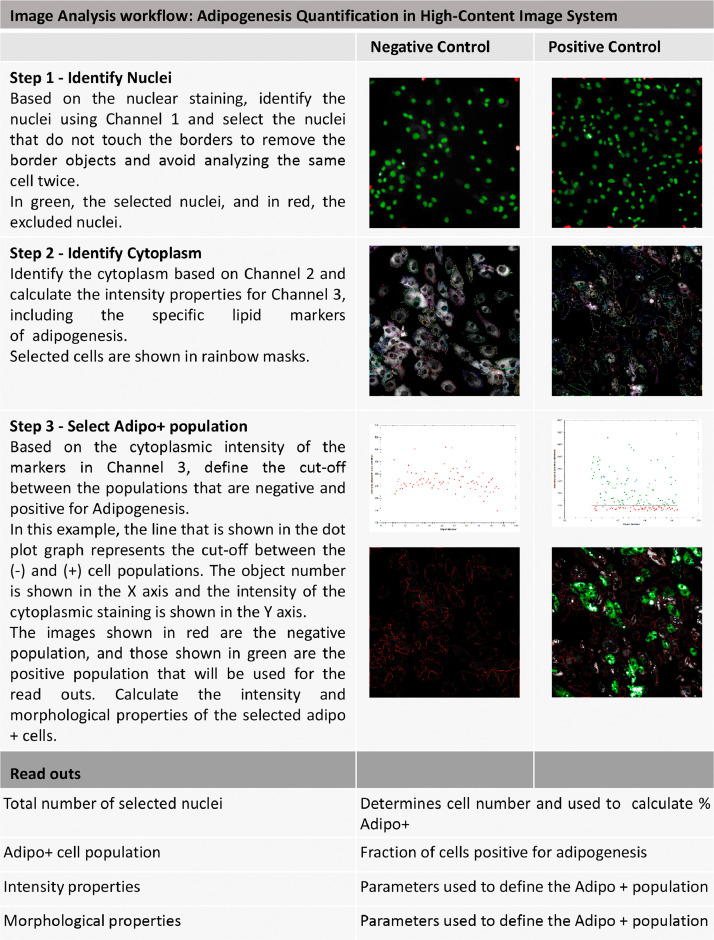
Image analysis workflow adipogenesis quantification with a high-content imaging system. Negative and positive controls are shown in the three fluorescence channels necessary for the image setup analysis for adipogenesis quantification to guide the reader with images for each step. The first step is to select the nuclei, and the second step is to identify the cytoplasm and calculate its characteristics. The third step is to select the Adipo+ population. The fourth step is to select the results for nuclei number, % Adipo+ cells, sum area of Adipo+ cells, and sum intensity of Adipo+ cells to populate the results table.

60. After performing the serial dilution of the test substance, prepare two sterile 6-well plates. Use only 9 wells, and number these wells from 1 to 8. The 9th well will contain vehicle.

61. Add 1.980 ml** of the test substance DM to wells 1 to 8 and to an extra well for vehicle (DMSO).

62. Add 20 µl** from the microtube containing each test substance solution to the corresponding wells. It is essential that adequate homogenization be performed after the addition of the test item solution to the wells to properly mix the test item with the previously added test item DM. Add 20 µl** of DMSO to the extra well containing 1.980 µl** of the test substance dilution medium (vehicle control).

NOTE: **The user can adjust these volumes as long as the proportion of 1:100 is maintained.

*NOTE: The addition of the 6-well plate serial dilution to the 96-well plates is the same as that demonstrated for the hydrosoluble test substance.*

### Test external positive control plate

The suggested external test control substance is SDS (see section Background information).

The suggested dilution factor (DF) is 1.47, according to Annex C, and we describe the details of the assay planning below.

63. Before starting the dilutions, SDS must be weighed and/or diluted to obtain a working solution [2x] based on the highest concentration tested. This information must be present in the planning document.

64. Vortex the solution for 1 to 2 min to ensure solubilization.

65. Similar to the hydrosoluble test substance, separate the two sterile 6-well plates. Use only 9 wells, and number these wells from 1 to 8.

66. Add 200 µg/ml of previously prepared working solution of SDS to well 1.

67. Add 2.35 ml of the test substance DM to wells 2 through 8, according to a DF of 1.47, as indicated in the planning document (Annex C).

68. Initiate the serial dilution from well 1, which contains 200 µg/ml SDS solution. Sequentially transfer 5 ml from wells 1 to 8. Perform adequate homogenization for each well to properly mix the test substance with the previously deposited medium.

*NOTE: Test the external positive control plate in parallel with the other test substances assayed by this method.*

*NOTE: The addition of the 6-well plate serial dilutions to the 96-well plates is the same as that demonstrated for the hydrosoluble test substance.*

## Declaration of Competing Interest

The authors declare that they have no known competing financial interests or personal relationships that could have appeared to influence the work reported in this paper.

## Figures and Tables

**Table 1:** Planning the assay. The linear and logarithmic values are shown below. We suggest using logarithm-based serial dilutions to determine the 8 concentrations that will be applied in the experiment. Exploratory assays may use a DF of 10 or 3.16 to embrace 8 and 4 log units, respectively. Once the range of effects is defined, other DFs may be applied, depending on the amplitude of the area of the curve to be evaluated. DF=dilution factor.

Number Scale	Linear						Logarithm					
DF	10	3.16	2.15	1.78	1.47	1.21	10	3.16	2.15	1.78	1.47	1.21
Concentration 1	1000	1000	1000	1000	1000	1000	3	3	3	3	3	3
Concentration 2	100	316.5	465.1	561.8	680.3	826.4	2	2.5	2.7	2.7	2.8	2.9
Concentration 3	10	100.1	216.3	315.6	462.8	683	1	2	2.3	2.5	2.7	2.8
Concentration 4	1	31.7	100.6	177.3	314.8	564.5	0	1.5	2	2.2	2.5	2.8
Concentration 5	0.1	10	46.8	99.6	214.2	466.5	-1	1	1.7	2	2.3	2.7
Concentration 6	0.01	3.2	21.8	56	145.7	385.5	-2	0.5	1.3	1.7	2.2	2.6
Concentration 7	0.001	1	10.1	31.4	99.1	318.6	-3	0	1	1.5	2	2.5
Concentration 8	1E-04	0.3	4.7	17.7	67.4	263.3	-4	-0.5	0.7	1.2	1.8	2.4
Number of Logs Screened							8 log	4 log	3 log	2 log	1 log	>1 log
Suggested Type of Assay							Exploratory Assay		Final Assay			

**Table 2:** The estimated starting dose for the ATC method was determined by the adipogenic inhibition assay to reduce the number of animals. The acute toxicity estimate (ATE), based on the LD_50_ value predicted by the adipogenic inhibition assay, can be used to estimate the starting dose for the ATC method in accordance with the classification of the GHS [32]. Data are shown for the next dose lower than the estimated LD_50_ in the default dose progression of 5, 50, 300, or 2000 mg/kg for the 2000 mg/kg limit test [10]. The minimum estimated starting dose was established as the suggested starting dose for the ATC method.

GHS Category*	Best Fit Starting Dose (mg/kg)
ATE ≤ 5 mg/kg (category 1)	5
5 < ATE ≤ 50 mg/kg (category 2)	5
50 < ATE ≤ 300 mg/kg (category 3)	50
300 < ATE ≤ 2000 mg/kg (category 4)	300
2000 < ATE ≤ 5000 mg/kg (category 5)	2000
ATE > 5000 mg/kg (unclassified)	2000

⁎ Globally Harmonized System of Classification and Labeling of Chemicals [34].

**Fig. 9:**
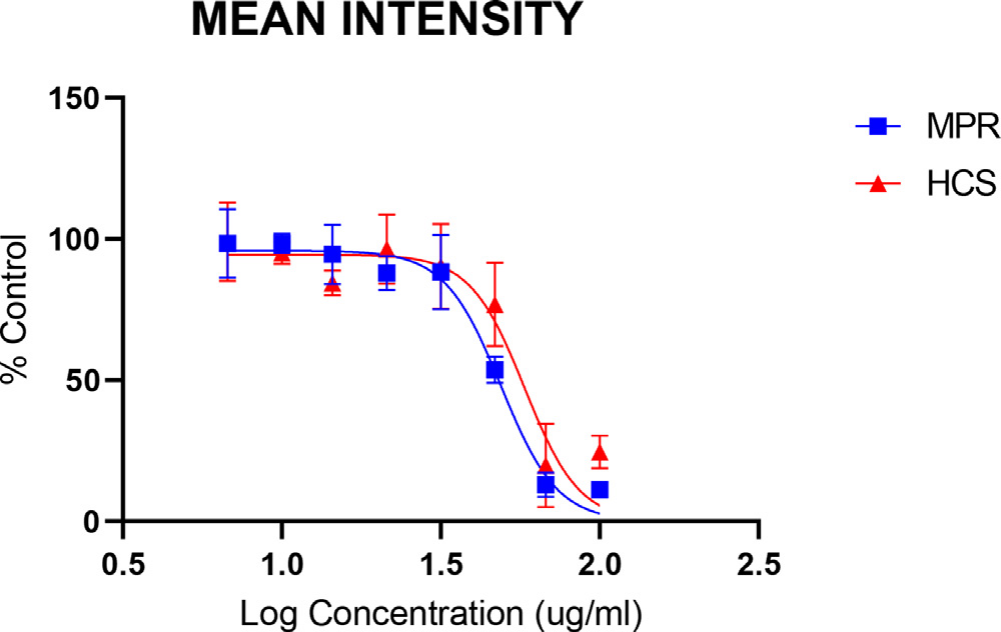
Comparing a multiplate reader and a high-content imaging system. A dose-response curve of the effects of SDS on the inhibition of adipogenesis using the protocol described here is shown. Dose-response curves are shown for the parameter intensity data obtained with the multiplate reader (MPR) and the high-content imaging system (HCS), and the overlay of the curves shows the agreement between the two readouts.

**Table 3:** Summary of the data for SDS-mediated inhibition of adipogenesis. Data obtained with a multiplate reader (MPR) and high-content imaging system (HCS) in 2 independent experiments are shown. The IC_50_ values are shown in µg/ml and mM, and these values were used to predict the LD_50_ values with Eq. (1) (RC rat-only weight regression: log LD_50_ (mg/kg) = 0.372 log IC_50_ (μg/ml) + 2.024) and Eq. (2) (RC rat-only millimole regression: log LD_50_ (mmol/kg) = 0.439 log IC_50_ (mM) + 0.621). R^2^ values were above 0.85, SSMD-Robust values were above 3, and the predicted GHS categories are shown.

Replicate	IC_50_ (µg/ml)	IC_50_ (mM)	LD_50_ (1)	LD_50_ (2)	R^2^	SSMD	Predicted GHS Category	Readout
1	21.29	6140.35	329.69	383.82	0.99	4.63	4	MPR
2	57.17	16487.07	476.07	592.16	0.96	4.10	4	
1	57.51	16583.55	477.10	593.68	0.98	16.92	4	HCS
2	77.65	22391.99	533.49	677.34	0.86	14.90	4	

**Table 4:** Troubleshooting Guide for the AD-MSC model for the *in vitro* modulation of adipogenesis applied to estimating the starting doses for an acute oral toxicity test.

Problem	Possible Solution
Cell plating is not homogeneous	Homogenize the cell solution before plating.
Volatile test items	Volatile test items have not been tested to date. If necessary, it is possible to use a permeable adhesive membrane.
Precipitate	If precipitates are observed in the serial dilutions, continue with the test and make the appropriate observations. It will be necessary to be stricter in the solubility test. If precipitates are observed in some wells, it is recommended that these samples not be used.
Contaminated cultures	This is an unusable assay. Discard the assay and verify the solutions for cell contamination before starting a new assay.
Failure in data acquisition by high-content imaging microscopy	Maintain the plates at room temperature minutes before acquisition to avoid tarnish. Do not leave fingerprints on the plates.
Failure in data acquisition with a hybrid microplate	Rupture any bubbles before starting plate data acquisition. Do not leave fingerprints on the plates.
Observation of an intact cell monolayer	Maintain smooth handling during plating and processing. If the monolayer is broken, it is recommended to discard the assay.
High standard deviation between the positive internal controls	Attention is required during plating, and it should be as homogeneous as possible. Homogenize the cell solution frequently.
Ambiguous results during data analysis	Review and alter the dilution interval, observe the curve and plan dilutions that explore the range from 0 to 100% effect.

**Table:** 

NaCl	80 g
KCl	4 g
Na_2_HPO_4_·12H2O	3.9 g
KH_2_PO_4_	1.5 g

**Table:** 

Glucose	11 g

**Table:** 

Phenol red	0.5 g

**Table:** 

Solution A	50.0 ml
Solution B	50.0 ml
Solution C	0.25 ml
Solution D	50.0 ml
Trypsin	0.5 g
EDTA	0.2 g

## Additional information

The protocol requires users to obtain AD-MSCs. AD-MSCs can be acquired from a commercial source (e.g., Lonza®, Walkersville, USA; catalog number PT-5006) or isolated according to standard protocols [18] from tissue collected during liposuction procedures after approval of the local ethics committee and donor informed consent. AD-MSCs need to be characterized [19,20] and maintained at 37°C ± 1°C, 90% ± 10% humidity and 5% ± 1% ambient CO_2_ until reaching confluence (80–90%). Then, the cells need to be subcultured and expanded. Cell characterization must be carried out according to the criteria recommended by Cell Therapy Society; these criteria include the ability to adhere to plastic, the potential to differentiate, and the expression or absence of recommended cell surface markers as determined by immunophenotyping data [19,20].

Second, it is necessary to know the physical and chemical characteristics of the test substance before starting these protocols. It is recommended to perform a solubility test on the test substance, as described by Stokes and collaborators [21]. After the solubility test, users should perform an exploratory test to determine the effects of each substance during the adipogenic differentiation process and construct an initial dose-response curve. Then, in a second test, users can repeat the assay using a narrower range of concentrations, with the goal of determining the best dose-response curve possible, including concentrations on the top and bottom and at least one concentration on the linear portion of the curve above 50% and below 50% differentiation. The exploratory test uses DFs of 10 or 3.16 for serial dilutions of the eight concentrations evaluated in this protocol (see below). These DFs are considered exploratory because within the eight points evaluated, the concentrations are quite broad, such as eight log intervals for DF 10 and 4 log intervals for DF 3.16. After exploratory experiments, the experiment can be refined by choosing narrower DFs that explore the region of interest, always including the minimum to maximum values of adipogenesis inhibition. Fig. 1 shows an example of an exploratory and final assay; as stated, the final assay uses the information from the exploratory assay to test a narrower range of concentrations.

The experimental design should be based on guideline 129 of the Organization for Economic Cooperation and Development (OECD) [12,21]. The planning stage should include the preparation of the test substance at all concentrations to be tested, and the experimental process in the laboratory should be clearly described in detail. Dilutions should cover all effect ranges (from no effect to maximum effect, with at least two dilutions at the latter level). Because the result plot (nonlinear regression based on the Hill function; see Statistical Analysis section) displays the concentration axis on a log scale, the dilutions themselves should also follow log scale factors. Table 1 shows the recommended test dilutions.

In addition, this test has internal and external controls that guarantee the quality of the test and the results obtained. All assay plates should have internal controls. These controls correspond to nondifferentiated and untreated cells (columns 1 and 12) and differentiated but untreated cells (columns 2 and 11). A plate with SDS, known to interfere with the differentiation process (Abud et al., 2019), is included to evaluate the test performance in all assays as an external test control.

In this protocol, the test plates are scanned with a high-content imaging system (e.g., Operetta CLS®, PerkinElmer®, Waltham, MA, USA, Image Xpress® Micro, Molecular Devices®, San Jose, CA, USA or similar) to provide morphological data related to the cell number and differentiation area, intensity, or percentage. Alternatively, users can acquire the data with a hybrid microplate reader (H1 M, BioTek®, Winooski, VT, USA or similar). The aim of this analysis is to obtain data on adipogenesis differentiation at wavelengths of 450–500 nm excitation and higher than 528 nm emission [13], [14], [15].

The use of animals for toxicity assessment has been questioned worldwide, and the 3R strategy is based on reducing, replacing, or refining their use, as first mentioned by Russell and Burch [22]. While human stem cells play a notable role in regenerative medicine, as reviewed by [23], their characteristics, such as self-renewal and differentiation potential, have also been exploited for toxicity prediction as a substitute for animal testing [24], [25], [26].

A well-known alternative method to animal testing in toxicology research is to use BALB/c 3T3 (murine fibroblast) or NHK (normal human keratinocytes) cell lines in the neutral red uptake (NRU) cytotoxicity assay to determine IC_50_ values and estimate LD_50_ values. This method is a validated *in vitro* cytotoxicity test that can estimate starting doses for acute oral systemic toxicity, as recommended by the OECD as test guidance no. 129 [12]. However, 3T3 cells are not human cells, and NHK cells require many culture media supplements, resulting in a higher cost to perform the test than regular cell lineages. To address these issues, in 2011, Scanu and colleagues concluded that bone marrow mesenchymal stem cells were similar to NHKs and murine BALB/c 3T3 fibroblasts by an NRU assay and could similarly be used to predict toxicity [5]. These results were very promising once easily maintained adult multipotent human cells cultured *in vitro* were utilized as cell substrates for toxicity prediction. However, because an invasive procedure is used to collect bone marrow-derived stem cells, obtaining stem cells from other sources would be highly beneficial.

Our group has previously shown that AD-MSCs have a sensitivity similar to that of BALB/c 3T3 cells when analyzed by the NRU assay [17]. These results showed that the use of both cell types accurately predicted substances with lower toxicity [17], although both failed to predict the correct GHS category for more toxic test substances [17]. A major concern worldwide is the poor correlation between *in vitro* and in vivo data. After our group obtained the abovementioned results, we questioned whether the ability of stem cells to differentiate could provide a methodology that would result in more accurate data to assess toxicity *in vitro* [4]. We chose adipogenesis because it is a very well-known, easily quantifiable cell differentiation model; thus, adipogenesis is more economically viable than pluripotent cell models, such as cardiac differentiation. This method makes it possible to use cells of human origin, and our previous data indicate that evaluating the toxic effects during the adipogenic differentiation process is more sensitive with relative viability assays, similar to traditionally used methods, such as the NRU assay [12]. We evaluated 10 test substances with a high-content imaging system, and the data provided by this test indicated that the inhibition of adipogenic differentiation predicted the toxicity of highly toxic substances more accurately than the NRU assay [4]. Thus, we suggest the use of this test as an alternative method to the use of animals that provides complementary results to the NRU assay.

In our previous study [4], we developed an assay quality control system that compares negative and positive internal controls according to the quality control criterion. We evaluated three quality control criteria: the Z’ factor; strictly standardized mean difference (SSMD); and robust SSMD. While the Z’ factor is the most commonly used quality control criterion, we chose the robust SSMD because of its better performance and certain statistical properties. The robust SSMD does not rely on the normality assumption, and it is quite insensitive to outliers because it uses the median instead of the mean. For these reasons, robust SSMD is better suited for cytotoxicity assays.

We also describe the test acceptance criteria to consider whether the test is valid, which includes the use of internal and external controls. Quantifying the differences between the positive and negative controls in each plate is a means by which to evaluate the quality of the assay. In our previous study [4], we developed an assay quality control system that compares negative and positive internal controls according to the quality control criterion of the robust strictly standardized mean difference (robust SSMD) approach.

In Abud et al. (2019), we established and validated SDS as an external positive control in an adipogenic inhibition assay. The performance external control is a separate plate of varying SDS concentrations. We used SDS because it is a substance with low toxicity (GHS category 4), is water soluble and shows good results in the adipogenic differentiation protocol; specifically, SDS toxicity is exhibited by a reduction in adipogenic differentiation compared with that of control cells. SDS affects cell count parameters and inhibits adipogenic differentiation in a concentration-dependent manner. This approach included the dose-response curves of cell differentiation that were generated using the Hill function and exhibited an R^2^ value ≥ 0.85, and agreement was shown among the resulting IC_50_ values, the predicted LD_50_ values, and estimated starting dose according to the ATC method, such as category 4 [27].

The ATC method can be applied to replace the traditional LD_50_ method [28], thereby reducing animal use [4]. If a previous method is applied to the ATC method, to suggest a better starting dose than the default starting dose of 300 mg/kg recommended when no previous information about the test substance toxicity is available [27], it is possible to reduce animal use. In a previous study, we applied the inhibition of adipogenesis method to predict the starting dose for the ATC method, with GHS categories 1 and 2 predicting the best fit starting dose of 5 mg/kg and category 3 predicting the best fit for the ATC method of 50 mg/kg [4].

Another interesting result was the improved accuracy of this method, which was assessed by comparing the IC_50_ values obtained with the parameters that assess cell numbers with the data obtained with the parameters that assess differentiation itself (% and area). The IC_50_ values that were obtained with the differentiation parameters were low for most substances tested, but these data became clearer when substances with high toxicity, such as cycloheximide and phenylthiourea, were evaluated [4]. Thus, this proposed methodology could be used in a complementary way to OECD n° 129 [12], which is an assay that better predicts substances with lower toxicity, according to the GHS categorization. Another study [4] demonstrated that it was possible to verify the hypothesis that this method would reduce the number of animals used. For this purpose, the researchers predicted the LD_50_ values based on the IC_50_ values obtained with the differentiation parameters; then, the initial dose was estimated for the ATC method.

Despite its many advantages, this strategy is a new method based on the effects exerted by substances on stem cell differentiation, which has not yet been validated in different laboratories. This method may be an alternative to animal use and for regulatory purposes after validation. Additionally, researchers could perform other approaches, such as a comparison of the data generated by this method and by animal repeated-dose assays.

In summary, researchers can use the protocol described here to evaluate cytotoxicity during cell differentiation processes *in vitro* and for other applications, such as the study of the basic biology of stem cells or the screening of test substances that may interfere with adipogenesis [29], [30], [31].

## Understanding the results

With this method, we aim to obtain dose-response curves based on the inhibition of adipogenic differentiation of induced AD-MSCs to determine the IC_50_ values that can predict LD_50_ values. We also aim to use this value to reduce the numbers of animals used by estimating the starting dose for the ATC test. Without performing a previous *in vitro* assay, the initial dose used in an in vivo assay was 300 mg/kg animal weight. A previous *in vitro* assay may indicate a lower starting range. In this manner, it is possible to reduce the number of animals used in the assay. The R^2^ values of the rat-only weight regression and rat-only millimole regression (0.372 and 0.452) were extremely low, meaning that their predictive power was poor. RC rat-only weight regression normally applies to mixtures in which the test substances are combined with others of unknown molarity, while RC rat-only millimole regression normally applies to pure substances [32]. Predicted LD_50_ values were used for all the analyzed parameters (nuclei, % differentiation, area differentiation) and in combination. We are proposing two readouts: one readout uses an approach based on a high-content imaging system to assess certain parameters related to cell differentiation, and the other readout uses multiplate reader analysis. As you can see from the data generated for the external positive control SDS in Fig. 9, both approaches provide similar dose-response curves for the intensity parameter. The IC_50_ values were calculated and used to predict the LD_50_ values that correspond to category 4 GHS, as summarized in Table 3; Table 3 also includes the R^2^ and SSMD values. Therefore, it is possible to obtain results suitable for LD_50_ prediction based on the intensity parameter with both readouts, depending on equipment availability.

Using a microplate reader (measuring the fluorescence intensity), it was possible to predict the toxicity of the substances (data not shown, manuscript in preparation). These data are interesting because microplate readers are less expensive pieces of equipment and are found more often in different laboratories.

However, a high-content imaging system can provide additional details and information, including the cell number, area and/or intensity of cell differentiation, the % cell differentiation and many other specific parameters. For this reason, a high-content imaging system is the first choice of equipment for this type of analysis, and morphological evaluation and cell counting data can also be considered as previously described [4,8].

The acceptance criteria of the test are as follows:

|SSMD_robust_| values ≥ 3 indicate that positive and negative control groups are different [33].

GraphPad Prism® software was able to calculate R^2^ for nonlinear regression. We advise the use of the R^2^ parameter to assess the goodness of fit of the regression curves. Its statistical suitability for nonlinear regression remains controversial. Nevertheless, R^2^ is widely required by regulatory guidelines when using sigmoidal curves in biological assays. Experiments that provide sigmoidal curves with an R^2^ value ≤ 0.85 [12] should be rejected, as this indicates a lack of fit of the data to the regression curve. However, the cutoff of 0.85 is arbitrary.

## Reagents and solutions

**Adipogenic differentiation induction medium (2X):** DMEM (e.g., Gibco Thermo Fischer®, Carlsbad, CA, USA - catalog number 12100046) supplemented with 10% fetal bovine serum (e.g., Gibco Invitrogen®, Carlsbad, CA, USA - catalog number 12657029), 4 mM L-glutamine (e.g., Gibco Invitrogen®, Carlsbad, CA, USA - catalog number 21051024), 100 units/ml penicillin and 100 µg/ml streptomycin (e.g., Gibco Invitrogen®, Carlsbad, CA, USA - catalog number 15140122), and adipogenic inducers, including 2 μg/ml insulin (e.g., Sigma-Aldrich®, St. Louis, MO, USA – catalog number I6634), 2 μM dexamethasone (e.g., Sigma-Aldrich®, St. Louis, MO, USA – catalog number D2915), 400 μM indomethacin (e.g., Sigma-Aldrich®, St. Louis, MO, USA – catalog number I7378), and 1 mM 3-isobutyl-1-methylxanthine (IBMX) (e.g., Sigma-Aldrich®, St. Louis, MO, USA – catalog number I5879). Store the stock solution at 2 to 8°C.

## Adipogenic differentiation inducers (stock solutions)

***Dexamethasone*** (5 ml of a 10 mM solution)

Molecular weight: 392.464 g/mol

Weigh 19.625 mg and dissolve in 5 ml of distilled water.

Filter through a 0.22-µm membrane.

Aliquot and store at –20°C.

***Insulin*** (20 ml of a 1 mg/ml solution)

Molecular weight: 5733.49 g/mol

Weigh 20 mg and dissolve in 20 ml of distilled water.

Add 50 µl of phenol red.

Adjust the pH to approximately 7.2 sterile NaOH 1 M or sterile HCl 1 M.

Filter through a 0.22-µm membrane.

Aliquot and store at –20°C.

***Indomethacin*** (20 ml of a 50 mM solution)

Molecular weight: 357.77 g/mol

Weigh 357.6 mg and dissolve in 20 ml of absolute ethanol.

Incubate in a water bath at 37°C for 30 min.

Vortex until completely dissolved.

Filter through a 0.22-µm membrane.

Aliquot and store at –20°C.

***IBMX*** (500 mM)

Molecular weight: 222.24 g/mol

Directly dilute IBMX with DMSO in the bottle.

To a 250-mg vial of IBMX, add 2.250 ml of DMSO.

Vortex until completely dissolved.

Filter through a 0.22-µm membrane.

Aliquot and store at –20°C.

*Maintenance medium for cell culture:* DMEM (e.g., Gibco Invitrogen®, Carlsbad, CA, USA - catalog number 12100046) supplemented with 10% fetal bovine serum (e.g., Gibco Invitrogen®, Carlsbad, CA, USA - catalog number 12657029) and 4 mM L-glutamine (e.g., Gibco Invitrogen®, Carlsbad, CA, USA - catalog number 21051024) without antibiotics. Store the stock solution at 2 to 8°C.

*Nile red solution:* Prepare a stock solution at a concentration of 1 mg/ml. To prepare this solution, 10 mg of Nile red (e.g., Sigma-Aldrich®, St. Louis, MO, USA – catalog number: N3013) must be weighed and diluted with 10 ml of DMSO (e.g., Sigma-Aldrich®, St. Louis, MO, USA – catalog number: D2650). Store the stock solution at room temperature.

*Paraformaldehyde solution* (4%): First, 20 g of paraformaldehyde (e.g., Sigma-Aldrich®, St. Louis, MO, USA – catalog number: 158127) must be weighed and dissolved in 200 ml of water preheated to 65°C under agitation (120 rpm). To dissolve this completely, slowly add approximately 2 ml of sodium hydroxide (1 M) or enough volume to make the solution clear. After this process, incubate the solution at room temperature and allow it to cool. Then, add 50 ml of PBS (10X) and adjust the pH to 7.4. Finally, adjust the volume to 500 ml with distilled water. Perform the entire process in a chemical fume hood. Store the stock solution at -20°C.

## PBS without calcium and magnesium [10X] (PBS 10X)

Sodium chloride (NaCl) (e.g., Sigma-Aldrich®, St. Louis, MO, USA – catalog number: S5886) - 80 g

Potassium chloride (KCl) (e.g., Sigma-Aldrich®, St. Louis, MO, USA – catalog number: P5405) - 2 g

Disodium phosphate (Na_2_HPO_4_) (e.g., Sigma-Aldrich®, St. Louis, MO, USA – catalog number: S5136) - 9.1 g

Monopotassium phosphate (KH_2_PO_4_) (e.g., Sigma-Aldrich®, St. Louis, MO, USA – catalog number: P5655) - 2 g

Adjust the volume to 1 L with distilled water.

Sterilize by autoclaving and store the stock solution at 2–8°C.

## PBS [1x]-Working solution

PBS [10X] - 100 ml

Sterile distilled water - 900 ml

Store the stock solution at 2–8°C.

*Test item dilution medium:* DMEM (e.g., Gibco Invitrogen®, Carlsbad, CA, USA - catalog number 12100046) supplemented with 4 mM L-glutamine (e.g., Gibco Invitrogen®, Carlsbad, CA, USA - catalog number 21051024) and 100 units/ml penicillin and 100 µg/ml streptomycin (e.g., Gibco Invitrogen®, Carlsbad, CA, USA - catalog number 15140122). Store the stock solution at 2 to 8°C.

*Trypan blue solution (0.4%) in sterile PBS* (e.g., Sigma-Aldrich®, St. Louis, MO, USA – catalog number: T6146).

## Trypsin/EDTA buffer

Prepare the 20X stock solutions for the Trypsin/EDTA Buffer.

**Solution A: 20X**

Adjust the volume to 500 ml with distilled water.

Sterilize by autoclaving and store the stock solution at 2–8°C.

**Solution B- 20x**

Adjust the volume to 500 ml with distilled water.

Sterilize by autoclaving and store the stock solution at 2–8°C.

**Solution C – 400X**

Adjust the volume to 200 ml with distilled water.

Dissolve in alkalized water with 1 N NaOH and adjust to the proper volume.

Sterilize by filtration through a 0.22 µm membrane. Store the stock solution at 2–8°C.

**Solution D – 20X**

Na_2_SO_4_ 1 g

Adjust the volume to 500 ml with distilled water.

Sterilize by autoclaving and store the stock solution at 2–8°C.

**Trypsin/EDTA solution:** Prepare a 0.05% trypsin solution (e.g., Sigma-Aldrich®, catalog number: T4799) with 0.02% EDTA (e.g., Sigma-Aldrich®, catalog number: E5134) in a suitable buffer.

Preparation of the trypsin/EDTA solution:

Adjust the volume to 1 L with sterile distilled water.

Adjust the pH to 7.6–7.8. Sterilize the solution by filtration through a 0.22-µm membrane. Store at -20°C.

## Appendix B Supplementary materials

Image, application 1

Image, application 2

Image, application 3
