# Supplementary material for: Using inhibition of the adipogenesis of adipose-derived stem cells in vitro for toxicity prediction
Source: MethodsX. 2021 Sep 14;8:101515. doi: 10.1016/j.mex.2021.101515 (PMC8564732; doi:10.1016/j.mex.2021.101515)
Supplement: Supplementary file 1 [file mmc1.docx]

**Annex A**

Planning document - Hydrosoluble test item

| **ASSAY IDENTIFICATION:** | | | | | **DATE:** | | | | | |
| --- | --- | --- | --- | --- | --- | --- | --- | --- | --- | --- |
| **CELL TYPE:** | | | | | **ASSAY OPERATOR:** | | | | | |
| **TEST ITEM:** | | | | | **CONCENTRATION (mg/ml):** | | | | | |
| **TEST ITEM DILUENT:** | | | | | **CONCENTRATION OF STOCK SOLUTION:** | | | | | |
| **DILUTION FACTOR (DF):** | | | | | **CONCENTRATION OF WORKING SOLUTION:** | | | | | |
|  |  |  |  |  |  |  |  |  |  |  |
|  |  |  |  |  |  |  |  |  |  |  |
| **FINAL CONCENTRATIONS (DF: 1:3.16)** | | |  | **SERIAL DILUTION PREPARATION** | | | | | | |
| log | linear (µg/ml) | well |  | **DILUTION FACTOR (DF)** | **1:10** | **1:3.16** | **1:2.15** | **1:1.78** | **1:1.47** | **1:1.21** |
| 2 | 100 | 1 |  | **TEST ITEM [2x]** | 1 ml | 1 ml | 1 ml | 1 ml | 1 ml | 1 ml |
| 1.500312917 | 31.64556962 | 2 |  | **TEST ITEM DM** | 9 ml | 2.16 ml | 1.15 ml | 0.78 ml | 0.47 ml | 0.21 ml |
| 1.000625835 | 10.01442077 | 3 |  | ***NOTE: The volumes can be adjusted to avoid waste of sample and culture medium.*** | | | | | |  |
| 0.500938752 | 3.169120496 | 4 |  |  |  |  |  |  |  |  |
| 0.00125167 | 1.002886233 | 5 |  |  |  |  |  |  |  |  |
| -0.498435413 | 0.317369061 | 6 |  |  |  |  |  |  |  |  |
| -0.998122496 | 0.100433247 | 7 |  |  |  |  |  |  |  |  |
| -1.497809578 | 0.031782673 | 8 |  |  |  |  |  |  |  |  |
|  |  |  |  |  |  |  |  |  |  |  |
