# Supplementary material for: Using inhibition of the adipogenesis of adipose-derived stem cells in vitro for toxicity prediction
Source: MethodsX. 2021 Sep 14;8:101515. doi: 10.1016/j.mex.2021.101515 (PMC8564732; doi:10.1016/j.mex.2021.101515)
Supplement: Supplementary file 2 [file mmc2.docx]

**Annex B**

Planning document - Nonhydrosoluble test item

| **ASSAY IDENTIFICATION:** | | | | | **DATE:** | | | | | | |
| --- | --- | --- | --- | --- | --- | --- | --- | --- | --- | --- | --- |
| **CELL TYPE:** | | | | | **ASSAY OPERATOR:** | | | | | | |
| **TEST ITEM:** | | | | | **CONCENTRATION (mg/ml):** | | | | | | |
| **TEST ITEM DILUENT:** | | | | | **CONCENTRATION OF STOCK SOLUTION:** | | | | | | |
| **DILUTION FACTOR (DF):** | | | | | **CONCENTRATION OF WORKING SOLUTION:** | | | | | | |
|  |  |  |  |  |  |  |  |  |  |  |  |
| **EXAMPLE OF NONHYDROSOLUBLE TEST ITEM** | | |  |  |  |  |  |  |  |  |  |
| **dilution factor: 1.78** |  |  |  |  |  |  |  |  |  |  |  |
| log | linear (µg/ml) | well |  |  |  |  |  |  |  |  |  |
| 3 | 1000 | 1 |  | **SERIAL DILUTION PREPARATION - dilution factor: 1.78** | | | | |  |  |  |
| 2.749579998 | 561.7977528 | 2 |  | **example of suitable proportion** | | | | |  |  |  |
| 2.499159995 | 315.6167151 | 3 |  | **TEST ITEM [2x]** | 1 ml | **100 µl*** | 50 µl | 25 µl |  |  |  |
| 2.248739993 | 177.3127613 | 4 |  | **DMSO (diluent used)** | 0.78 ml | **78 µl** | 39 µl | 19.5 µl |  |  |  |
| 1.998319991 | 99.61391083 | 5 |  | *** proportion chosen for the assay** | |  |  |  |  |  |  |
| 1.747899988 | 55.96287125 | 6 |  |  |  |  |  |  |  |  |  |
| 1.497479986 | 31.43981531 | 7 |  | 1) Prepare a stock solution (tube 1). Example of a stock solution – 200,000 µg/ml. | | | | |  |  |  |
| 1.247059984 | 17.66281759 | 8 |  | 2) Perform serial dilutions in microtubes numbered 2 to 8 and add 78 µl of DMSO. | | | | | |  |  |
|  |  |  |  | 3) Add 100 µl of the solution from the previous tube. | | |  |  |  |  |  |
|  |  |  |  | 4) Add 2.970 µl** of the test item dilution medium to wells 1 to 8 and to an extra well for the vehicle (DMSO). | | | | | | | |
|  |  |  |  | 5) Add 30 µl** of the microtube test item solution to the wells. | | | |  |  |  |  |
|  |  |  |  | 6) Add 30 µl** of DMSO to the extra well containing 2.970 µl** of the test item dilution medium (vehicle control). | | | | | | | |
|  |  |  |  | ** These volumes can be adjusted, as long as the proportions are maintained. | | | | |  |  |  |
| **NOTE 1: For test items that are soluble in DMSO, the final concentration of the vehicle applied to the cells should be a maximum of 0.5% (v/v), both in the vehicle control and in the 8 concentrations used (11).** | | | | | | | | | | | |
|  |  |  |  |  |  |  |  |  |  |  |  |
| **NOTE 2: For test items that are soluble in DMSO, concentration 1 (well 1) should contain 1/200 of the highest concentration considered to be soluble in the solubility test (15); in this case, concentration 1 is the stock solution.** | | | | | | | | | | | |
|  |  |  |  |  |  |  |  |  |  |  |  |
