# Supplementary material for: Using inhibition of the adipogenesis of adipose-derived stem cells in vitro for toxicity prediction
Source: MethodsX. 2021 Sep 14;8:101515. doi: 10.1016/j.mex.2021.101515 (PMC8564732; doi:10.1016/j.mex.2021.101515)
Supplement: Supplementary file 3 [file mmc3.docx]

**Annex C**

Planning document – External Positive Control

| **ASSAY IDENTIFICATION:** | | | | | **DATE:** | | | |
| --- | --- | --- | --- | --- | --- | --- | --- | --- |
| **CELL TYPE:** | | | | | **ASSAY OPERATOR:** | | | |
| **TEST ITEM: SDS** | | | | | **CONCENTRATION (mg/ml):** | | | |
| **TEST ITEM DILUENT:** | | | | | **CONCENTRATION OF STOCK SOLUTION: 2000 µg/ml** | | | |
| **DILUTION FACTOR (DF):** | | | | | **CONCENTRATION OF WORKING SOLUTION: 200 µg/ml (well 1)** | | | |
|  |  |  |  |  |  |  |  |  |
|  |  |  |  |  |  |  |  |  |
| **FINAL CONCENTRATIONS (DF: 1:1.47)** | | |  | **SERIAL DILUTION PREPARATION - DF: 1:1.47** | | |  |  |
| log | linear (µg/ml) | well |  | **TEST ITEM [2x]** | 1 ml | **5 ml*** |  |  |
| 2 | 100 | 1 |  | **TEST ITEM DM** | 0.47 ml | **2.35 ml** |  |  |
| 1.832682665 | 68.02721088 | 2 |  | *** proportion chosen for the assay** | |  |  |  |
| 1.665365331 | 46.27701421 | 3 |  | ***NOTE: The volumes can be adjusted to avoid waste of sample and culture medium.*** | | | | |
| 1.498047996 | 31.48096205 | 4 |  |  |  |  |  |  |
| 1.330730661 | 21.41562044 | 5 |  |  |  |  |  |  |
| 1.163413326 | 14.56844928 | 6 |  |  |  |  |  |  |
| 0.996095992 | 9.910509713 | 7 |  |  |  |  |  |  |
| 0.828778657 | 6.741843342 | 8 |  |  |  |  |  |  |
|  |  |  |  |  |  |  |  |  |
